# Influence of the Synergistic System of Carbon-Based Fillers with Melamine Polyphosphate on the Thermal Properties and Fire Hazard of Flexible Polyurethane Foams

**DOI:** 10.3390/ma19020267

**Published:** 2026-01-08

**Authors:** Arkadiusz Głowacki, Przemysław Rybiński, Witold Żukowski, Anna Zawierucha, Ulugbek Zakirovich Mirkhodjaev, Monika Żelezik

**Affiliations:** 1 Institute of Chemistry, The Jan Kochanowski University, 25-406 Kielce, Poland; annazawierucha.011@gmail.com; 2 Faculty of Chemical Engineering and Technology, Cracow University of Technology, Warszawska 24, 31-155 Cracow, Poland; witold.zukowski@pk.edu.pl; 3 Department of Biophysics, National University of Uzbekistan, Tashkent 100174, Uzbekistan; u.z.mirkhodjaev@gmail.com; 4 Institute of Geography and Environmental Sciences, The Jan Kochanowski University, 25-406 Kielce, Poland; monika.zelezik@ujk.edu.pl

**Keywords:** PUR composites, smoke emission, fire hazard, carbon based fillers, thermal properties

## Abstract

In the article we investigated the effectiveness of a synergistic system designed to reduce the fire hazard of flexible polyurethane (PUR) foams. The examined system consisted of a carbon-based filler graphene (G), carbon nanotubes (CNTs), or expanded graphite (EG) combined with melamine polyphosphate (MPP). The investigated polyurethane foams (PUR) were synthesized at room temperature via a polycondensation reaction between a polyol and an isocyanate, with an OH: NCO molar ratio of 2:1. Both the carbon fillers and melamine polyphosphate were homogeneously dispersed within the polyol component. Thermogravimetric analysis (TGA), cone calorimetry, and microcalorimetry were used to evaluate the influence of the fillers on the thermal stability and flammability of the PUR foams. The toxicity of the gaseous products was assessed using a coupled TG-gas analysis system, while the optical density of the evolved gases was determined using a Smoke Density Chamber (SDC). The obtained results demonstrated that the applied synergistic carbon-phosphorus filler system significantly reduced the fire hazard of the tested PUR foams. In particular, the EG5-MPP system enabled the formation of self-extinguishing materials.

## 1. Introduction

Polyurethane (PUR) materials have found widespread use in everyday life and across numerous industrial sectors, including the automotive and construction industries, where they serve, among others, as insulation and cushioning materials [1,2]. The broad applicability of polyurethane-based materials results from their excellent physicochemical properties, such as low thermal conductivity, low density, high abrasion resistance, and good energy absorbing capability. Moreover, PUR materials can be produced on a large scale using relatively simple processing methods. One of the key advantages of PUR systems is their high susceptibility to modification, which can be achieved by altering the type of raw materials, adjusting the reactant ratio, introducing fillers or modifiers, or modifying the processing conditions. Depending on the chemical composition and synthesis route, the resulting composites may exhibit high mechanical strength, enhanced vibration and noise damping, increased resistance to physicochemical factors, or improved resistance to organic solvents and oils [3,4,5].

Despite numerous advantages, polyurethane materials also exhibit several significant limitations. A major drawback of PUR foams which severely restricts their applicability, particularly in the automotive and construction industries, is their high flammability and the rapid kinetics of their thermal degradation [6]. The combustion of PUR foams is accompanied by substantial heat release, dense smoke formation, and the generation of toxic decomposition products, primarily carbon monoxide (CO) and carbon dioxide (CO_2_). At raised fire temperatures, i.e., above 400 °C, hydrogen cyanide (HCN) and nitrogen oxides (NO_x_) are also released. These toxic compounds can cause severe damage to the respiratory, nervous, and cardiovascular systems. In extreme cases, exposure to high concentrations of fire effluents generated during the thermal decomposition and combustion of PUR materials may lead to fatal outcomes [7,8,9].

The literature increasingly reports synergistic flame-retardant systems, often referred to as hybrid flame retardant formulations, which combine the advantages of different filler groups to impart new, frequently unique properties to polymer matrices. PUR foams are increasingly being modified using cage-like fillers (POSS), often in combination with phosphorus-based compounds, metal phosphinates, or various inorganic mineral fillers [10,11,12]. Widely studied mineral fillers include metal hydroxides such as Al(OH)_3_ and Mg(OH)_2_, aluminosilicates, hydroxyapatites, and metal phosphates [13,14]. The flame-retardant mechanism of inorganic compounds is based primarily on physical heat absorption during thermal decomposition and subsequent release of water vapor, which both limits oxygen access and dilutes the combustible gas phase. Furthermore, mineral fillers often promote the formation of a ceramic residue, which acts as a protective barrier effectively insulating the material from the heat source [15,16].

Another important group of fillers comprises carbon-based materials. Due to their ability to form three-dimensional networks within the polymer matrix, these fillers significantly influence the mechanical properties and thermal stability of composites [17,18]. The most well-known representatives include linear carbon nanostructures such as carbon nanotubes (CNTs) and carbon nanofibers. These materials are characterized by high mechanical strength, excellent electrical and thermal conductivity, and relatively low density [19,20]. Numerous studies have reported the effective use of CNTs to enhance the performance of polymers such as polyethylene, polystyrene, polylactic acid (PLA), and poly(vinyl alcohol) (PVA) [21,22,23].

Expanded graphite and its derivatives, including graphene, represent another important class of carbon fillers. Incorporating graphene into a polymer matrix leads to the formation of layered structures in which single- or few-layer graphene sheets act as physical barriers limiting gas diffusion. Such composite systems are characterized by high electrical conductivity, excellent thermal conductivity, and remarkable mechanical strength. Additionally, a single graphene layer is essentially impermeable to gases, making graphene one of the most promising carbon fillers for the development of polymeric barrier materials and functional composites [24,25].

The selection of fillers in polymer composites represents a critical stage in the development of polymeric materials, as their appropriate choice and dosage directly affect both the functional and processing properties. An excessively low additive content may fail to provide the desired functional effect, whereas an excessive loading can lead to deterioration of the material morphology and degradation of its performance characteristics. In the case of carbon-based fillers, it is particularly important to maintain a balance between the barrier effect and the risk of particle agglomeration, which may disrupt the foaming process [26]. The scientific literature indicates that, to achieve the desired physicochemical properties without inducing adverse effects, filler loadings typically range from a few weight percent to values often exceeding 20 wt.%, depending on the type of filler employed. The concentrations are selected to enable the flame-retardant action in the condensed phase through the formation of a stable char layer, while simultaneously minimizing the negative impact on the mechanical properties and flexibility of the foams [27].

The aim of the present study was to improve the performance of flexible polyurethane foams. In this work, PUR foams were modified using carbon-based fillers, including carbon nanotubes, expanded graphite (EG), and graphene (G), both individually and in combined systems with melamine polyphosphate (MPP). The article presents results on the influence of the combined system composed of a carbon filler and melamine polyphosphate (MPP) on the surface morphology, thermal stability, and flammability of flexible PUR foams. Particular attention was devoted to smoke emission and the toxicity of gaseous fire effluents formed under controlled thermal degradation and combustion conditions.

## 2. Material and Methods

### 2.1. Materials

The subject of the study was flexible polyurethane foam (PUR). The PUR foams were prepared via a one-step synthesis method using a polyol—diethanolamine (BASF, Elastoflex W5165/140) and an isocyanate component—diphenylmethane diisocyanate (MDI) (BASF, IZO 135/158). Catalysts and blowing agents were pre-incorporated into the polyol by the manufacturer. As fillers, expanded graphite (EG), graphene (G), carbon nanotubes (CNTs), and melamine polyphosphate (MPP) were used.

#### Preparation of PUR Composites

The PUR foams were prepared via a polycondensation reaction between a polyol and an isocyanate at an OH:NCO ratio of 2:1. The unmodified flexible PUR foam was obtained using a one-step synthesis carried out at room temperature.

The polyol and isocyanate components were introduced into the reaction vessel and mixed using a mechanical stirrer until intensive foam growth was observed. The foaming system was then transferred into a test form with a volume of 0.9 L. After completion of the foaming process, the resulting PUR foam was left in the open form for 60 min to allow stabilization of its physical structure.

**Figure 1:**
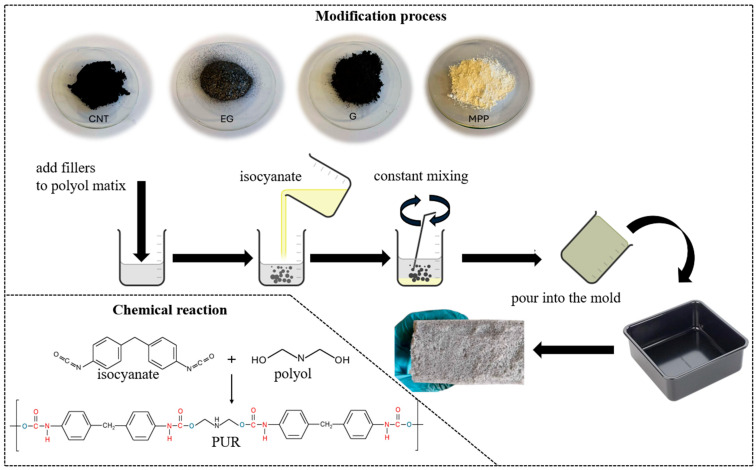
Schematic diagram of the PUR composite synthesis process.

The preparation of PUR composites was carried out in two stages (Figure 1). In the first stage, the fillers were introduced into the polyol in the appropriate proportions, in accordance with the information provided in Table 1. The polyol–filler mixture was stirred until the system became as homogeneous as possible. In the second stage, the synthesis process proceeded analogously to that of the unmodified PUR foam.

Before the experimental procedures, the prepared composites were conditioned to a constant mass at 23 ± 2 °C and a relative humidity not exceeding 50 ± 5%, in accordance with ISO 291 [28]. The conditioning process was considered completed when the sample mass showed no variation exceeding 0.1 g or 0.1% of the initial mass after 24 h.

### 2.2. Methods

#### 2.2.1. Fourier Transform Infrared Spectroscopy

Spectroscopic measurements were performed using Fourier transform infrared spectroscopy (FTIR) with a PerkinElmer Spectrum Two spectrometer (PerkinElmer, Waltham, MA, USA) equipped with a diamond ATR accessory mounted on a ZnSe plate. Data acquisition was carried out using Spectrum Software 10.03.06 (PerkinElmer, Waltham, MA, USA). Spectra were recorded in transmission mode within the mid-infrared (MIR) range of 400–4000 cm^−1^, at a resolution of 4 cm^−1^, using four scans per measurement.

#### 2.2.2. Scanning Electron Microscopy

Scanning electron microscopy (SEM) was performed using an Apero 2 S LoVac microscope (Thermo Fisher Scientific, Waltham, MA, USA) equipped with an energy dispersive X-ray spectroscopy (EDS) detector: UltraDry (Thermo Fisher Scientific, Waltham, MA, USA) and Octane Elect (EDAX) Ametek GmbH (Hitachi, Tokyo, Japan). All measurements were conducted at an accelerating voltage of 2 kV.

#### 2.2.3. Thermal Analysis

Thermal analysis of the fillers and PUR composites was carried out using an STA 449 F3 Jupiter thermogravimetric analyser (TGA) (Netzsch, Selb, Germany). The measurements were performed within the temperature ranges of 25–650 °C for PUR composites and 25–900 °C for fillers, using samples of 5 ± 1 mg placed in open Al_2_O_3_ crucibles. Analyses were conducted under an O_2_/N_2_ atmosphere (20/40 µL/min) at a constant heating rate of 10 °C/min. Thermograms were processed using Proteus Thermal Analysis 8.0.3 software (Netzsch, Selb, Germany). Based on the obtained TG and DTG curves, the following thermal parameters were determined: the temperature of 5% mass loss (T_5_), the temperature of 50% mass loss (T_50_), the temperature of maximum decomposition rate (TR_MAX_), the decomposition rate (dm/dt), the temperature range of char oxidation (ΔT_s_), the residue after thermal decomposition (P_TD_) and the residue at 600 °C (P_600_) and 900 °C (P_900_).

#### 2.2.4. Pyrolysis Combustion Flow Calorimetry

Flammability analysis of the materials was performed using a pyrolysis combustion flow calorimeter (PCFC, Fire Testing Technology Ltd., East Grinstead, UK) in accordance with ASTM D 7309 [29]. The pyrolizer temperature was set to 650 °C, and the combustor temperature to 900 °C. Measurements were carried out for samples of 5 ± 1 mg under a linear heating rate of 1 °C/s in an N_2_/O_2_ atmosphere (80/20 cc/min). During the analysis, the following parameters were recorded: the maximum heat release rate (HRR_MAX_, W/g), the temperature corresponding to HRR_MAX_ (T_HRR_), the total heat release (THR, kJ/g) and the heat release capacity (HRC, J/gK). These fire hazard parameters were determined using Fire Testing Technology software (FTT MCC ASTM 1.1).

#### 2.2.5. Cone Calorimetry

Fire behaviour under simulated real-scale fire conditions was evaluated using a cone calorimeter (Fire Testing Technology Ltd., East Grinstead, UK) in accordance with PN-EN ISO 5660 [30]. Samples with dimensions of 100 mm × 100 mm × 5 mm were placed horizontally and exposed to a heat flux of 35 kW/m^2^ generated by the cone heater. The analysis of fire hazard parameters was performed using MLCCalc 1.0.3 software (Fire Testing Technology Ltd., East Grinstead, UK). During the test, the following parameters were recorded: the time to ignition (t_i_), the time to flameout (t_f–0_), the heat release rate (HRR, kW/m^2^), the maximum heat release rate (HRR_MAX_, kW/m^2^), the time corresponding to HRR_MAX_ (tHRR_MAX_, s), the total heat release (THR, MJ/m^2^), the average effective heat of combustion (EHC, MJ/kg), the maximum effective heat of combustion (EHC_MAX_, MJ/kg), the mass loss rate (MLR, g/s), the maximum mass loss rate (MLR_MAX_, g/s), the average mass loss rate (AMLR, g/m^2^·s), the fire growth rate index (FIGRA, kW/m^2^·s) and the maximum average rate of heat emission (MARHE, kW/m^2^).

#### 2.2.6. Smoke Density Analysis

Smoke optical density was determined using the single-chamber test (Smoke Density Chamber, SDC) in accordance with PN-EN ISO 5659-2 [31]. Samples of 75 mm × 75 mm × 5 mm were exposed to a radiant heat flux of 25 kW/m^2^. The monitored parameters, recorded using SDCSoft 1.0.4.0 (Fire Testing Technology Ltd., East Grinstead, UK), included the maximum optical smoke density (D_sMAX_), the time to reach the maximum optical smoke density (TD_sMAX_), the optical smoke density at 4 min (Ds(4)) and the smoke obscuration area under the emission curve during the first four minutes of the test (VOF4). All recorded values are dimensionless, except for Ds(4), expressed in seconds.

#### 2.2.7. Toxicity Analysis

The analysis of toxic gaseous products was performed using a coupled analytical system comprising an Omega 5 Bruker gas analyzer (Bruker, Billerica, MA, USA) equipped with an FTIR spectrometer, connected to an F1 Libra 209 Netzsch thermogravimetric analyzer (TG) (Netzsch, Selb, Germany). Thermal decomposition of samples with masses of 10 ± 1 mg was conducted at three temperatures, 450 °C, 550 °C and 750 °C, in synthetic air with a flow rate of 40/20 µL/min (nitrogen/oxygen). The decomposition programs were set so that gases produced during thermal degradation were collected for 30 min, with a 15 min isothermal segment at each temperature. The gaseous products formed during decomposition were analyzed in real time using FTIR spectroscopy (Bruker, Billerica, MA, USA), enabling the recording of gas evolution as a function of temperature. Spectra were recorded at intervals of 7–8 s (10 scans), at a resolution of 4 cm^−1^, in the mid-infrared range (MIR) from 400 to 4500 cm^−1^, in transmission mode. The obtained spectral data were analyzed using OPUS GA 5.2.11.9 software (Bruker, Billerica, MA, USA), where absorbance values were converted into concentrations (ppm). Subsequently, based on Equation (1), the concentrations expressed in ppm were converted to values in g/m^3^.

(1) $$C=\frac{ppm\times M}{22,414}$$

where

C—is the concentration expressed in mg/m^3^.
M—is the molar mass expressed in g/mol.
22,414—is the molar conversion constant in dm^3^/mol.

Statistical analysis of toxicity and the calculation of the Fractional Effective Dose indices (FED, WLC_50_ and WLC_50SM_) were carried out in accordance with the asphyxiant toxicity model defined in ISO 13571 (Purser model) [32]. The FED index was determined by incorporating the toxic contributions of CO and HCN, together with the hyperventilation correction associated with CO_2_ concentration [32,33]. All calculations were based on the time-resolved gas concentration profiles obtained during the thermal decomposition of the PUR samples.

The hyperventilation correction factor, v, was applied following the ISO 13571 methodology. In the Purser model, this correction factor defined as a function of the instantaneous CO_2_ concentration is applied to all asphyxiant gas indices:

(2) $$v\left(t\right)=\left(\frac{{CO}_{2}\left(t\right)}{5}\right),\;\;if\;\;{CO}_{2}\left(t\right)>2\%$$

Whenever the condition CO_2_(t) > 2% was satisfied, the corresponding gas concentrations were corrected:

(3) $$CO\left(t\right)={CO\left(t\right)}^{*}*v\left(t\right)\;$$

(4) $$HCN(t)={HCN(t)}^{*}*v(t)$$

where the quantities HCN(t)* and CO(t)* refer to the concentrations prior to the application of the hyperventilation correction v.

Calculating the instantaneous dose model:

(5) $$FEDi=\sum_{t1}^{t2}\frac{CO}{35,000}\Delta ti+\sum_{t1}^{t2}\frac{\exp\left(\frac{HCN}{43}\right)}{220}\Delta ti$$

where

(6) $$\Delta t=t\left(i\right)-t(i-1)$$

Calculating the cumulative FED index:

(7) $$FED=\sum_{i=2}^{k}FEDi,\;\;\;\;FED\left(t1\right)=0$$

Calculation of the fractional contributions of CO and HCN to the FED index:

(8) $$FED\_CO\left(i\right)=\sum \frac{COi}{35,000}∆ti,\;$$

(9) $$FED\_HCN\left(i\right)=\sum \frac{\exp\left(\frac{HCN}{43}\right)i}{220}∆ti,\;$$

(10) $$FEDtot\left(i\right)=FED\_HCN(i)+FED\_CO(i)$$

(11) $$HCN\%i=\frac{FEDHCN\left(i\right)}{FEDtot(i)}$$

(12) $$\frac{CO}{HCN}i=\frac{CO\%i}{HCN\%i}$$

Determination of the FEC for the remaining irritant gases:

(13) $$FEC=\sum_{j}\frac{C_{j}\left(t\right)}{{IC}_{50j}}$$

where

C_j_(t)—the instantaneous concentration of gas j in ppm (HCl, HBr, HF, SO_2_, NO, NO_2_, acrolein, formaldehyde or other species).
IC_50j_—the threshold concentration of this gas (also referred to as LC_50_) in ppm, in accordance with ISO 13571.

The threshold values and corresponding exposure times (FED 0.3, FED 1.0 and FED30) were determined from the cumulative FED.

FED 0.3 is defined as the exposure time at which the FED index first reaches a value of 0.3 and is associated with the end of the safe exposure period to the gas mixture within the enclosure.

FED 1.0 is defined as the exposure time at which the FED index first reaches a value of 1.0, corresponding to the attainment of a critical toxic dose of gases in the enclosure.

FED30 denotes the FED value attained after 30 min of testing.

Additional linear interpolation of the FED indices was applied where necessary:

(14) $$t^{*}=ti-1+\frac{{FED}^{*}-FED\left(ti-1\right)}{FED\left(ti\right)-FED\left(ti-1\right)}(ti-ti-1)$$

where FED* denotes the interpolated FED value exceeding the threshold of 0.3 or 1.0.

Calculation of the toxicity of the tested materials.

(15) $$W{LC}_{50}=\frac{M\left(g\right)}{FED30*\;V\left(m^{3}\right)}$$

where

M—the mass lost during the test (initial mass minus final mass).
V—the effective exposure volume.
Calculation of the WLC_50_ toxicity index:
  (16) $${WLC}_{50SM}=\frac{1}{3}(WLC50_{T450}+WLC50_{T550}+WLC50_{T750})\;$$

## 3. Results and Discussion

### 3.1. Analysis of FTIR Spectra of the Fillers

**Figure 2:**
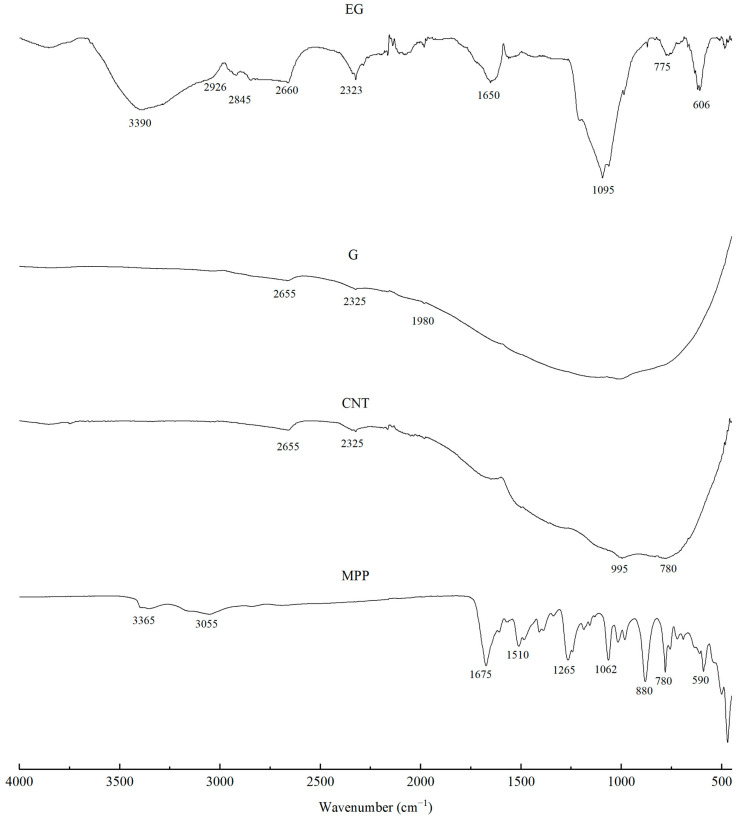
FTIR analysis of G, EG and CNTs and MPP fillers.

The FTIR spectra of the fillers, i.e., CNTs and G, showed high-intensity overlapping bands in the range of approximately 1700–600 cm^−1^, which indicates the presence of numerous functional groups characteristic of carbon-based fillers (Figure 2). The high absorbance in this spectral region may be attributed to a large contribution of stretching vibrations originating from C–C, C–O–C, C–H, C–OH, O–H, C=O, C=C groups, as well as vibrations associated with aromatic rings [34,35,36]. The bands at around 1890 cm^−1^ are assigned to C=C bonds in cyclic systems, those at 1685 cm^−1^ and in the range 730–665 cm^−1^ to C=C bonds in aliphatic systems, while the band at 1450 cm^−1^ corresponds to bending vibrations of C–H groups. The region 2000–1900 cm^−1^ is associated with asymmetric stretching vibrations of C=C=C and C=C bonds. Wavenumbers in the 1300–1000 cm^−1^ range are ascribed to bending vibrations of C–H groups, whereas the range 1600–1585 cm^−1^ is related to skeletal C–C stretching vibrations. Weak bands in the 2850–2960 cm^−1^ range most likely arise from stretching vibrations of methyl groups [37,38].

The spectrum of the EG filler is characterized by a broad band in the 3420–3380 cm^−1^ range, corresponding to O–H stretching vibrations. The bands at 2926 and 2845 cm^−1^ indicate the presence of methylene (C–H) groups, which are typical for the 2850–2960 cm^−1^ region. The bands observed between 1340 and 1700 cm^−1^ can be assigned to C=C bonds, whereas the broad band at 1640 cm^−1^ is associated with the presence of carboxyl groups and with C–H stretching vibrations. A characteristic peak at around 1095 cm^−1^ indicates the presence of C–O bonds in the chemical structure of the filler [39,40,41].

Analysis of the MPP filler spectrum reveals a broad band at approximately 3365 cm^−1^, corresponding to asymmetric stretching vibrations of NH_2_ groups typical of melamine. The peaks at 3160 cm^−1^ and 1673 cm^−1^ are also assigned to NH_2_ stretching and deformation vibrations, respectively. Vibrations around 3100 cm^−1^ correspond to O–H and C–H groups. The bands at 1510 cm^−1^, 1409 cm^−1^ and 757 cm^−1^ are ascribed to characteristic vibrations of the triazine ring in MPP [42]. In addition, the bands at 1406 cm^−1^ and 1017 cm^−1^ correspond to absorption of O–H groups. The bands at 1265 cm^−1^ and 880 cm^−1^ are attributed to P=O and P–O–P bonds, whereas the bands at 1242 and 1155 cm^−1^ are assigned to C–C stretching vibrations. The band at 1187 cm^−1^ is also associated with NH_2_ group vibrations [43,44].

### 3.2. Analysis of FTIR Spectra of PUR Composites

**Figure 3:**
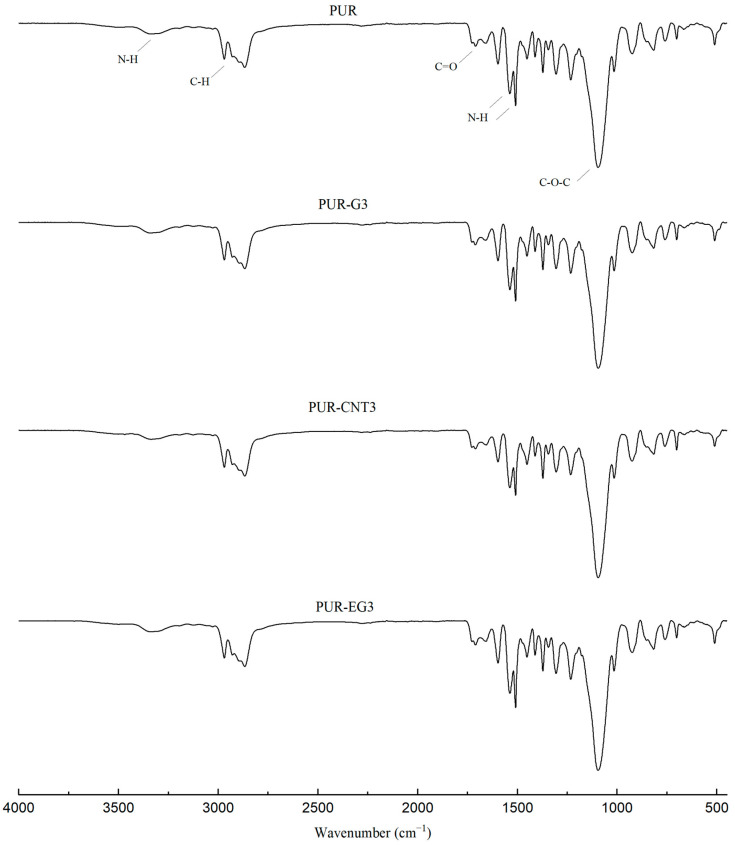
FTIR analysis of the PUR composites PUR, PUR-CNT3, PUR-G3 and PUR-EG3.

During the synthesis of flexible PUR foams, a characteristic disappearance of the band at approximately 2270 cm^−1^, assigned to the stretching vibrations of NCO groups, is typically observed. In the case of the PUR composites investigated in this study, a small absorption signal is still visible in this spectral region (Figure 3). This may suggest that the applied carbon-based fillers have the ability to shield NCO groups, which may affect both the chemical and mechanical properties of the resulting PUR composites.

**Figure 4:**
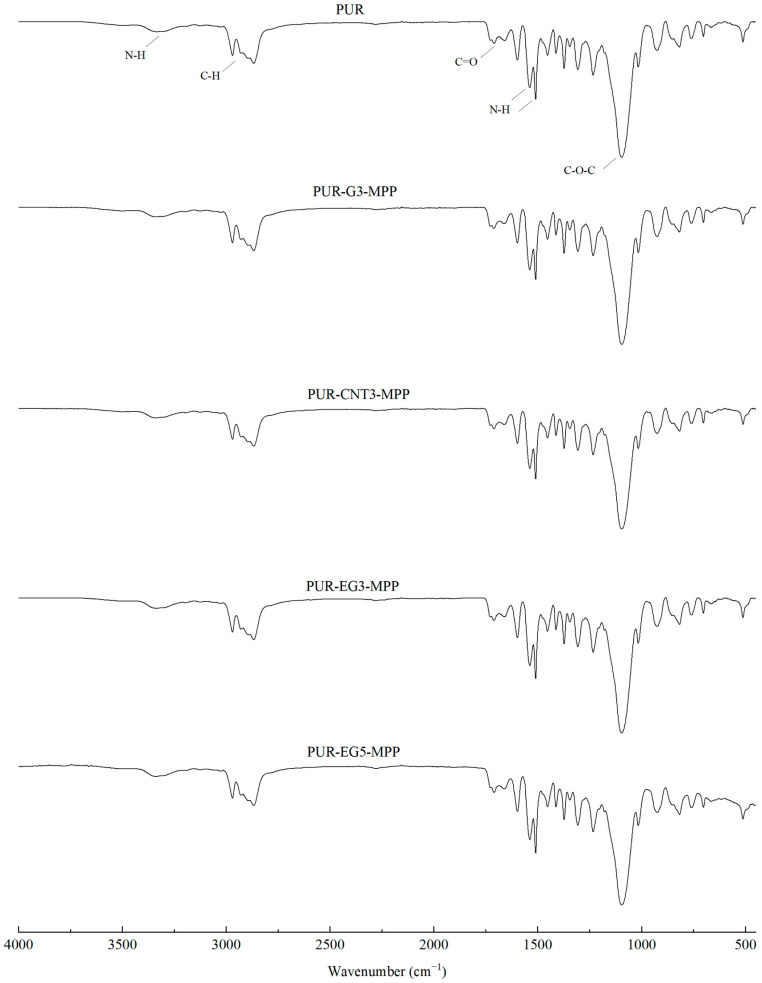
FTIR analysis of the PUR composites PUR-CNT3-MPP, PUR-G3-MPP, PUR-EG3-MPP and PUR-EG5-MPP.

In the FTIR spectra of the reference PUR foam, broad absorption bands are observed at approximately 3337 cm^−1^, as well as at 1460 cm^−1^ and 1260 cm^−1^, corresponding to the stretching vibrations of N–H groups. The absorption bands at 1460 and 1260 cm^−1^ can be attributed to the symmetric and asymmetric vibrations of N–C–N groups, respectively [45]. The bands at 2955 and 2851 cm^−1^ are characteristic of symmetric and asymmetric stretching vibrations associated with methylene groups present in the PUR foam chains. The bands at 1450 cm^−1^ are assigned to bending vibrations of C–H groups. In addition, the IR spectrum of the PUR matrix exhibits bands at 1655 and 1710 cm^−1^, which correspond to the stretching vibrations of carbonyl (C=O) groups. A distinct band at around 1095 cm^−1^ is characteristic of C–O–C stretching vibrations [46,47]. The incorporation of carbon fillers, including their synergistic combination with MPP, does not alter the overall character of the IR spectra (Figure 4).

### 3.3. SEM-EDS Analysis of the Fillers

Both the particle size distribution and the elemental composition of the flame-retardant additives play a crucial role in the mechanisms responsible for reducing the flammability of polymer composites. Carbon-based compounds used as fillers and modifiers in polymer matrices are widely applied not only to partially replace petrochemical components used in polymer synthesis, but also to enhance the physicochemical properties of the resulting materials.

**Figure 5:**
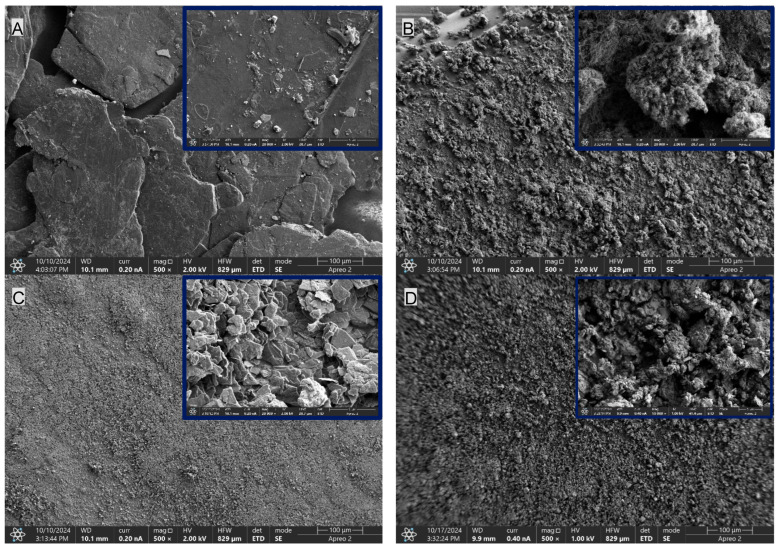
Comparison of the morphology of the PUR composite fillers: (**A**)—EG, (**B**)—CNTs, (**C**)—G and (**D**)—MPP. Imaging performed using SEM. The upper-right inset shows a 5 µm magnification.

The SEM images presented in Figure 5 show clear morphological differences between the analyzed fillers. The CNTs, G and MPP fillers exhibit pronounced granularity and irregular particle shapes, whereas the EG filler displays the smallest particle size and the presence of large, smooth plate-like structures with a more homogeneous morphology.

**Figure 6:**
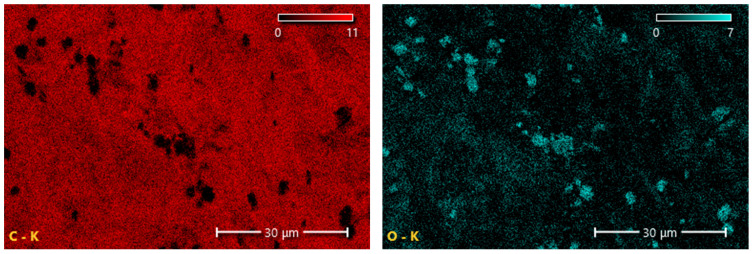
Carbon and oxygen distribution map of the EG filler obtained using SEM-EDS.

**Figure 7:**
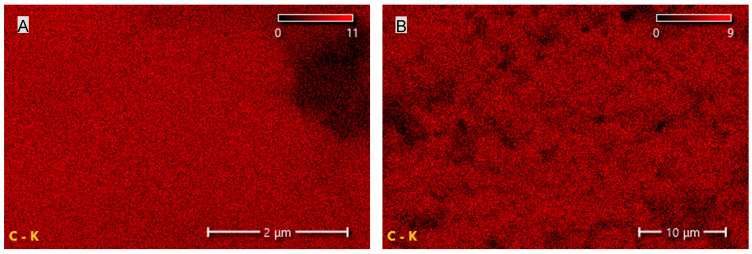
Carbon distribution maps of the CNT (**A**) and G (**B**) fillers obtained using SEM-EDS.

The results summarized in Table 2 and shown in Figure 6 and Figure 7 indicate that CNTs and graphene (G) exhibit the highest carbon content among the tested carbon-based fillers. This observation directly correlates with the findings from FTIR analysis, where a broad band characteristic of carbon–carbon bonds and hydrocarbon functional groups was recorded. In the case of EG, the carbon content is only 67.8%, which is 21.6% lower than that of CNTs and 31.8% lower than that of G. The reduced carbon content in EG compared with CNTs and G may negatively affect the uniformity and, consequently, the insulating performance of the protective boundary layer formed during the thermal degradation of PUR composites containing EG.

The MPP sample subjected to EDS analysis did not generate detectable elemental signals, which prevented its inclusion in the quantitative comparison (Table 2).

### 3.4. SEM-EDS Analysis of the PUR Composites

**Figure 8:**
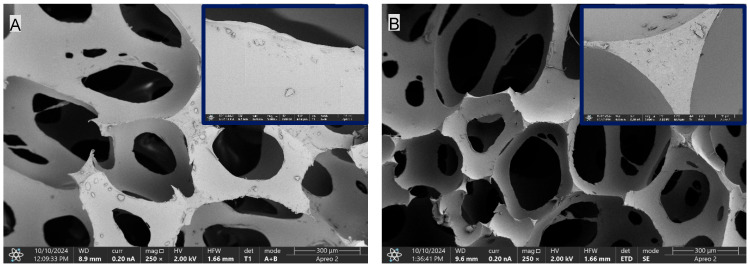

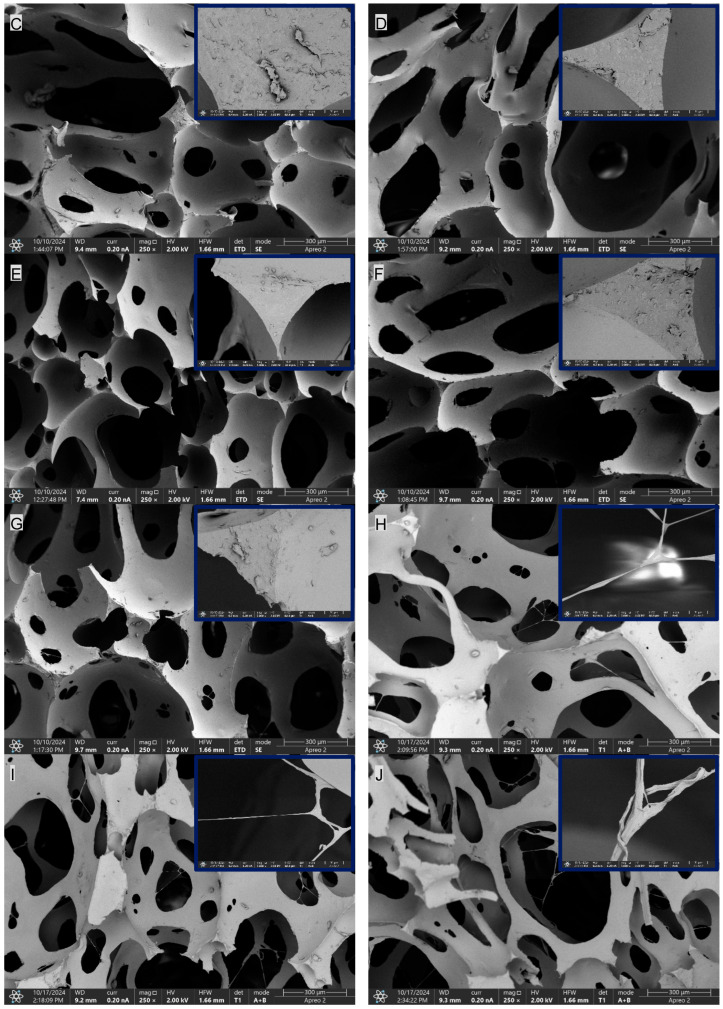
SEM imaging of the PUR composites: (**A**)—PUR, (**B**)—PUR-EG1, (**C**)—PUR-EG3, (**D**)—PUR-EG3-MPP, (**E**)—PUR-G1, (**F**)—PUR-G3, (**G**)—PUR-G3-MPP, (**H**)—PUR-CNT1, (**I**)—PUR-CNT3, (**J**)—PUR-CNT3-MPP. The upper-right inset shows a 10 µm magnification.

In the SEM images of the composites containing CNTs, namely PUR-CNT1, PUR-CNT3 and PUR-CNT3-MPP, the carbon nanotubes are clearly visible. By forming a three-dimensional spatial network within the polymer matrix, the nanotubes reduce the porosity of the analyzed composites (Figure 8).

**Figure 9:**
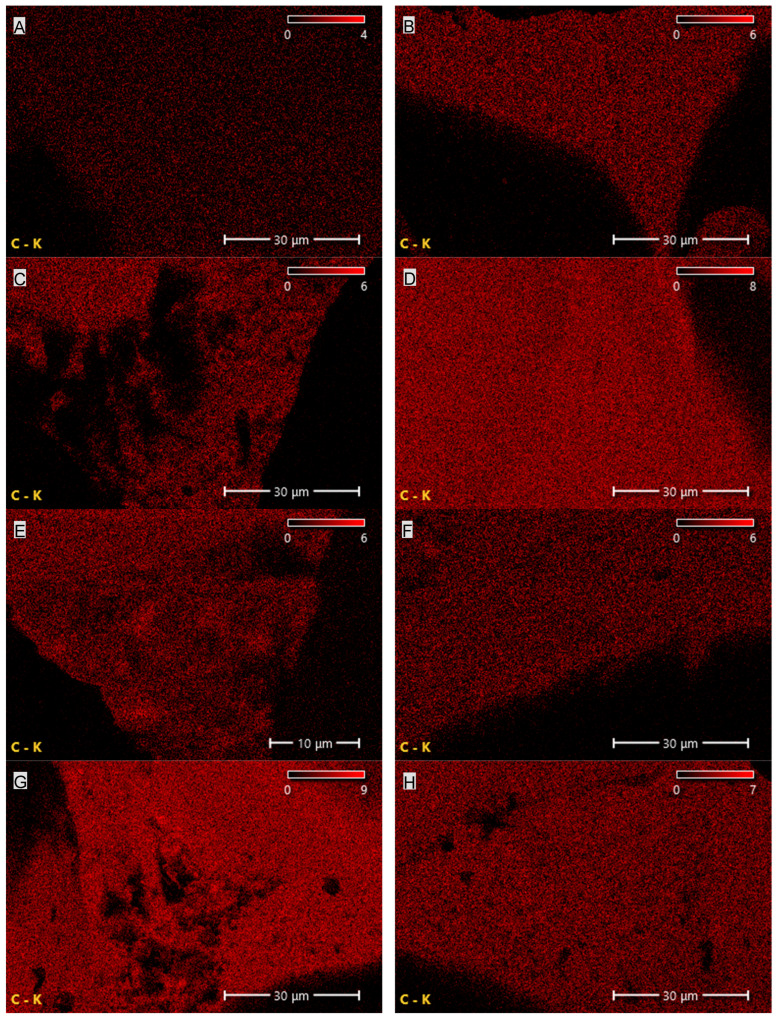

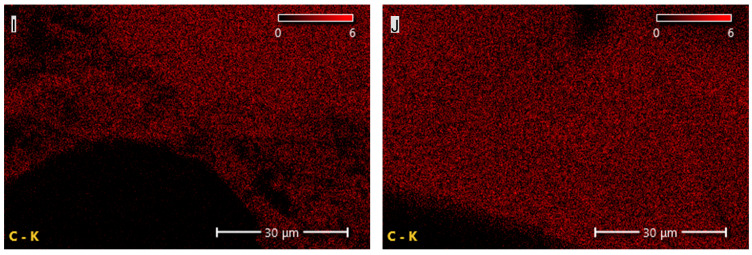
Elemental (carbon) distribution maps of the PUR composites: (**A**)—PUR, (**B**)—PUR-EG1, (**C**)—PUR-EG3, (**D**)—PUR-EG3-MPP, (**E**)—PUR-G1, (**F**)—PUR-G3, (**G**)—PUR-G3-MPP, (**H**)—PUR-CNT1, (**I**)—PUR-CNT3 and (**J**)—PUR-CNT3-MPP. Imaging obtained using the SEM-EDS technique.

**Figure 10:**
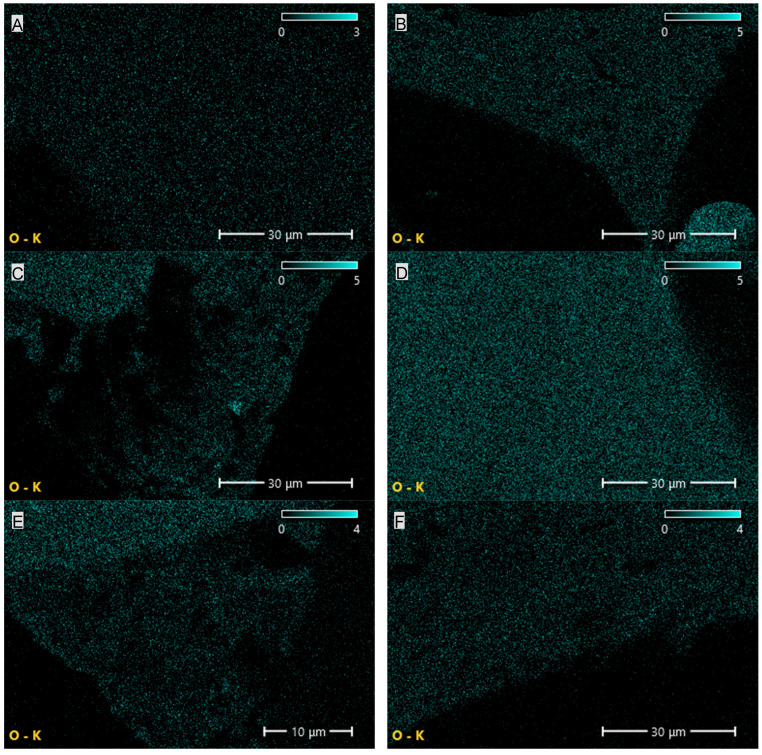

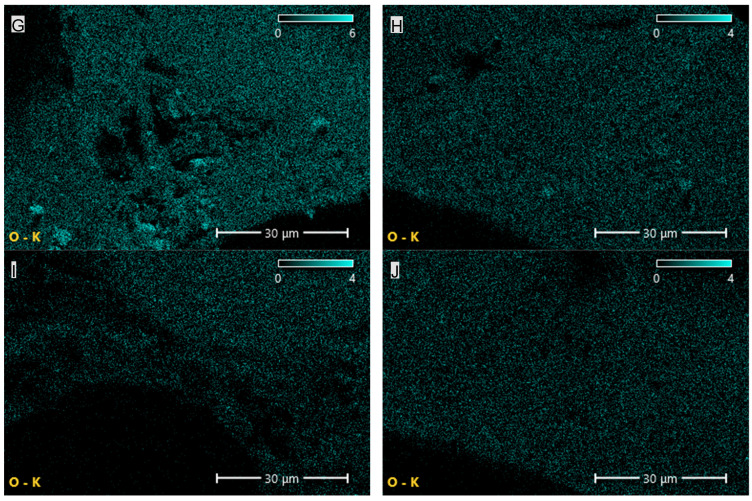
Elemental (oxygen) distribution maps of the PUR composites: (**A**)—PUR, (**B**)—PUR-EG1, (**C**)—PUR-EG3, (**D**)—PUR-EG3-MPP, (**E**)—PUR-G1, (**F**)—PUR-G3, (**G**)—PUR-G3-MPP, (**H**)—PUR-CNT1, (**I**)—PUR-CNT3 and (**J**)—PUR-CNT3-MPP. Imaging obtained using the SEM-EDS technique.

**Figure 11:**
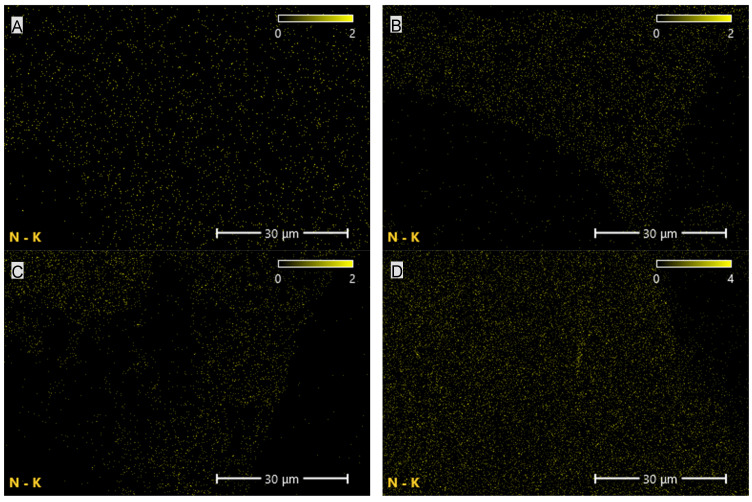

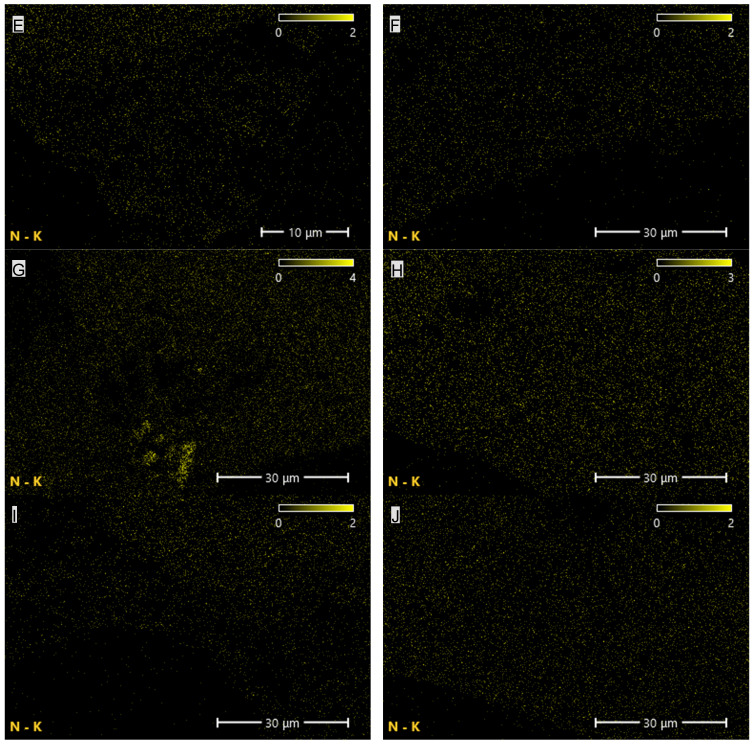
Elemental (nitrogen) distribution maps of the PUR composites: (**A**)—PUR, (**B**)—PUR-EG1, (**C**)—PUR-EG3, (**D**)—PUR-EG3-MPP, (**E**)—PUR-G1, (**F**)—PUR-G3, (**G**)—PUR-G3-MPP, (**H**)—PUR-CNT1, (**I**)—PUR-CNT3 and (**J**)—PUR-CNT3-MPP. Imaging obtained using the SEM-EDS technique.

The elemental composition of the PUR composites revealed the presence of carbon, nitrogen and oxygen in nearly uniform proportions, amounting, respectively, to 51.1–57.4%, 6.3–8.1% and 34.5–41% (Table 3; Figure 9, Figure 10 and Figure 11). The highest carbon and nitrogen contents were observed for the graphene-filled composites PUR-G1 and PUR-G3, whereas the highest oxygen content was recorded for the PUR-EG1 composite.

### 3.5. Thermal Analysis of the Fillers

**Figure 12:**
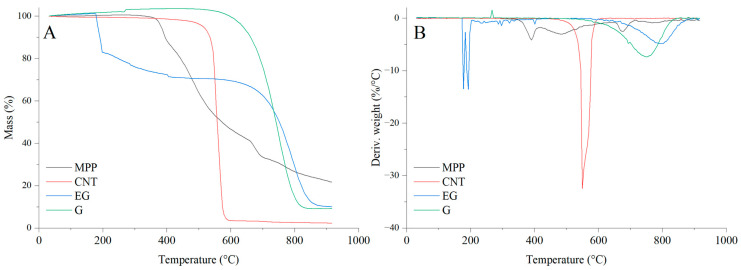
TG (**A**) and DTG (**B**) curves of the fillers used in the PUR composites.

The results of the thermal analysis of the investigated fillers clearly indicate that they exhibit markedly different thermal stabilities (Figure 12, Table 4). Among the analyzed fillers, graphene shows the highest thermal stability, as reflected by both the T_5_ and T_RMAX_ parameters. This filler undergoes a thermo-oxidative degradation, which begins above 620 °C (Figure 12), and it also exhibits the lowest decomposition rate (dm/dt) among all carbon-based fillers studied. Carbon nanotubes (CNTs), similar to graphene (G), also undergo a thermo-oxidative degradation; however, the CNTs filler displays significantly lower thermal stability, as indicated by T_50_ and T_RMAX_, compared with the other carbon fillers, i.e., G and EG. It is also notable that CNTs show a considerably higher decomposition rate and a lower amount of residue after thermal decomposition, as well as reduced residues at 600 and 900 °C, compared with G and EG.

In contrast to G and CNTs, expanded graphite (EG) exhibits a distinct two-stage mass-loss profile under oxidative conditions. The first stage, occurring in the temperature range of approximately ΔT = 180–200 °C, is attributed to a redox reaction between the intercalating agents and the graphite layers. This process is accompanied by the release of glowing gases, leading to the characteristic expansion of EG. The second stage, observed at higher temperatures above 600 °C, corresponds to the thermo-oxidative degradation of the graphite structure itself. Combustion of the residual char takes place within the ΔT = 650–850 °C range [48].

The thermal decomposition of MPP begins at 380 °C. Notably, MPP exhibits the slowest decomposition rate among all analyzed fillers. The residue remaining after decomposition at 600 and 900 °C amounts to 46.7% and 22.4%, respectively.

**Figure 13:**
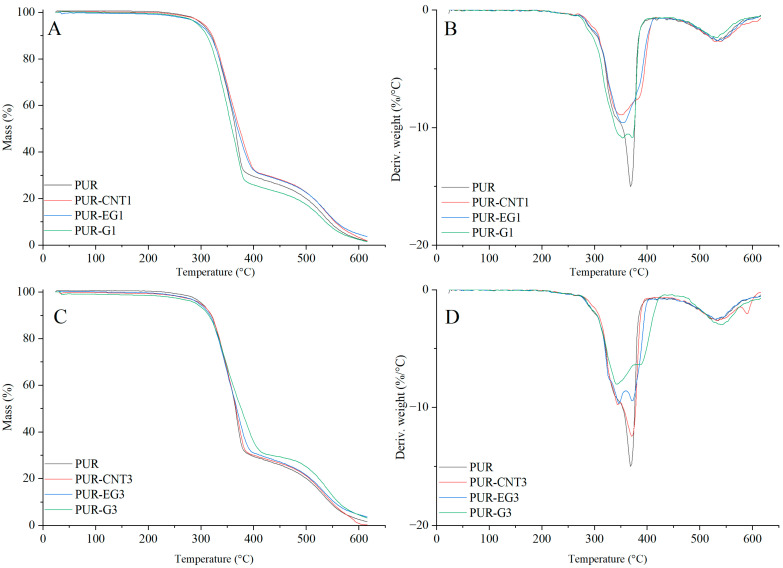
TG (**A**,**C**) and DTG (**B**,**D**) curves of the composites: PUR, PUR-CNT1, PUR-EG1, PUR-G1, PUR-CNT3, PUR-EG3 and PUR-G3.

**Figure 14:**
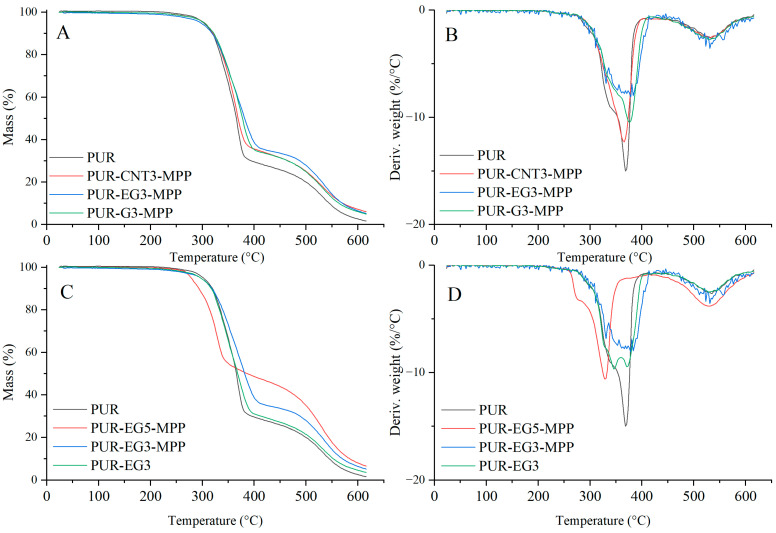
TG (**A**,**C**) and DTG (**B**,**D**) curves of the composites: PUR, PUR-CNT3-MPP, PUR-G3-MPP and PUR-EG3, PUR-EG3-MPP, PUR-EG5-MPP.

Based on the results of the thermal analysis of the PUR composites in an oxygen atmosphere (Table 5, Figure 13 and Figure 14), it was found that all of the analyzed materials exhibit a single-stage thermal decomposition process characteristic of flexible PUR foams, occurring within the temperature range ΔT = 25–400 °C. Combustion of the decomposition residue takes place in the ΔT = 400–600 °C range [49,50]. The results further showed that carbon-based fillers (CNTs, G and EG), when introduced into the polymer matrix at a loading of 1%, do not significantly influence the thermal stability of the PUR composites, as expressed by the T_5_, T_50_ or T_RMAX_ parameters. It should be noted, however, that all carbon fillers markedly reduce the dm/dt parameter. The reduction in dm/dt, which is proportional to the filler content within the polymer matrix, indicates that a smaller amount of volatile, combustible degradation products is released from the sample into the flame zone. The greatest reduction in dm/dt was observed for the PUR-G3 and PUR-EG3-MPP samples.

Almost all the tested fillers caused an increase in the amount of solid residue after thermal decomposition. The highest increase in P_TD_ reaching 34.6%, was recorded for the combined EG3-MPP system. Increasing the content of expanded graphite in the PUR-EG-MPP composite resulted in a proportional increase in the char residue, as observed for the PUR-EG5-MPP sample. The increase in the P_TD_ parameter for the combined carbon-filler/melamine-polyphosphate system arises directly from its mechanism of action. Melamine polyphosphate undergoes extensive foaming during thermal decomposition, forming a solid protective phase, whereas the carbon fillers act as carbon donors. In addition, the phosphoric acid released during the decomposition of MPP catalyzes the crosslinking reactions of the developing char layer [51,52,53].

### 3.6. Flammability Analysis

The flammability of flexible PUR foams is a critical factor determining their overall fire hazard. In assessing the fire behavior of polymeric materials, key importance is attributed to parameters such as the heat release rate (HRR), total heat released (THR), time to ignition (tᵢ), time to flame-out (t_f–0_), fire growth rate (FIGRA) and the maximum average rate of heat emission (MARHE). These parameters collectively describe both the intensity of combustion and the total energy released during the burning process.

Analysis of the data presented in Table 6 shows that PUR composites containing only carbon-based fillers (CNTs, G, EG) exhibit a pronounced reduction in fire hazard parameters compared with the unfilled PUR foam. The results clearly demonstrate that the reduction in flammability parameters is directly proportional to the amount of carbon fillers incorporated into the polymer matrix. Considering the HRR and MARHE values, the highest flame-retardant effectiveness is observed in the following order: expanded graphite, graphene and carbon nanotubes. In the presence of 3% expanded graphite, the HRR_MAX_ parameter was reduced by 64%, whereas the same loading of graphene and CNTs resulted in reductions of 27% and 22.6%, respectively. The MARHE parameter decreased by 59%, 17.2% and 4.5% for 3% EG, G and CNTs, respectively.

It is also important to emphasize that the applied carbon-based fillers significantly reduce the mass loss rate (MLR) of the analyzed composites (Table 6). The MLR values correlate well with the dm/dt values obtained from thermal analysis.

The use of a combined flame-retardant system MPP combined with graphene, expanded graphite or carbon nanotubes substantially reduces the flammability of the PUR foams. Among the investigated flame-retardant systems, the most effective was the combination of expanded graphite and melamine polyphosphate (MPP). For the PUR-EG3-MPP composite, reductions of 71% in HRR_MAX_, 76% in FIGRA and 74.5% in MARHE were observed. In this composite, a significant extension of the time to flame-out (61%) relative to the reference sample was also recorded. It should be noted, however, that the time to ignition, as in the case of other tested composites, was considerably shortened.

**Figure 15:**
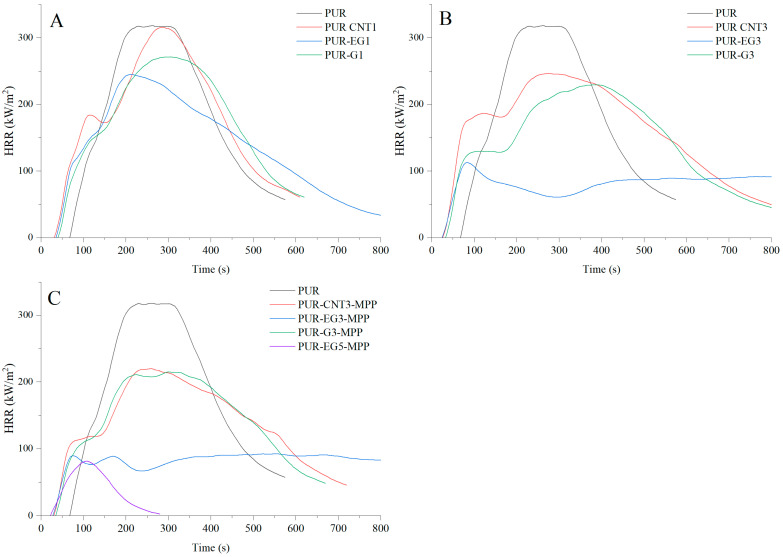
Heat release rate (HRR) profiles expressed in kW/m^2^ for the PUR composites: (**A**)—carbon-based composites with a concentration of 1%, (**B**)—carbon-based composites with a concentration of 3%, (**C**)—carbon-based composites with MPP. The analysis were recorded using the cone calorimeter at a radiant heat flux of 35 kW/m^2^.

**Figure 16:**
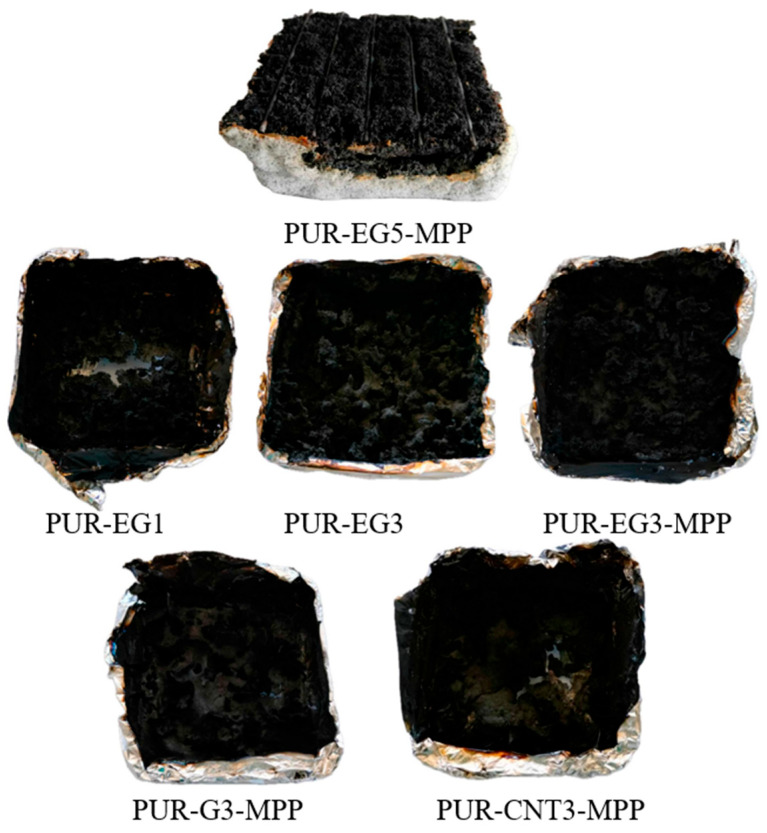
Residues remaining after cone calorimetry testing.

Increasing the content of expanded graphite from 3% to 5% (sample PUR-EG5-MPP) led to a further reduction in HRR_MAX_, FIGRA and MARHE (Table 7). Moreover, this sample exhibited a self-extinguishing effect, with the material extinguishing after only 154 s from the moment of ignition (Figure 15). This phenomenon is particularly important from a fire safety perspective, as the limitation of heat release coupled with rapid flame extinction can significantly reduce the risk of fire propagation, which is especially critical in the case of highly flammable flexible PUR foams (Figure 16).

The flammability results obtained using pyrolysis combustion flow calorimetry (PCFC) are presented in Table 8. The fire hazard parameters determined under conditions approximating real-scale scenarios using the cone calorimeter exhibit the same trend of reduced heat release rates for the PUR-G3-MPP, PUR-CNT3-MPP, PUR-EG3-MPP and PUR-EG5-MPP composites. These materials showed significant reductions in HRR_MAX_, reaching 388, 389, 375 and 341 W/g, respectively, compared with the reference PUR sample (552.6 W/g). It is also worth noting that the tested flame-retardant systems led to a marked decrease in the total heat release (THR), with values of 21.4 kJ/g for PUR-G3-MPP, 20.5 kJ/g for PUR-CNT3-MPP, 21.6 kJ/g for PUR-EG3-MPP and 21.6 kJ/g for PUR-EG5-MPP.

**Figure 17:**
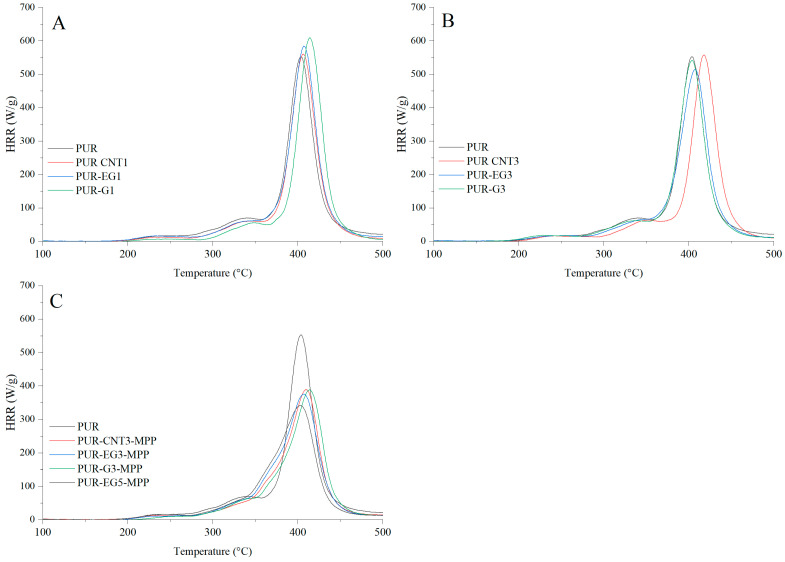
Heat release rate (HRR) curves expressed in W/g for the PUR composites: (**A**)—carbon-based composites with a concentration of 1%, (**B**)—carbon-based composites with a concentration of 3%, (**C**)—carbon-based composites with MPP. The analysis were obtained using PCFC microcalorimetry.

The HRR curves as a function of time for the composites containing carbon-based fillers combined with MPP are presented in Figure 17. The obtained results reveal a common feature for all analyzed samples. In the temperature range ΔT = 280–380 °C, thermo-oxidative degradation of the sample occurs, while in the range ΔT = 380–450 °C the combustion of volatile degradation products takes place. The introduction of carbon fillers, particularly G and EG, clearly reduces the HRR value (Figure 17A,B). For all combined systems incorporated into the PUR matrix, a significant reduction in the HRR parameter was recorded (Figure 17C).

### 3.7. Optical Smoke Density

In addition to thermal stability, flammability and heat emission, another critical parameter in evaluating the fire hazard of polymeric materials is the smoke produced during thermal decomposition and combustion. Smoke is defined as an aerosol of solid or liquid particles generated during incomplete combustion and pyrolysis. It consists of solid particulates, condensed-phase products, liquid mists and mixtures of volatile organic and inorganic compounds, which may be toxic, irritant or corrosive. The extent of smoke generation depends on various factors, including the chemical composition of the material, oxygen availability, heat flux intensity and combustion conditions (flaming or non-flaming). Smoke formation is also influenced by the carbon content of the material’s chemical structure, its degree of aromaticity, the presence of unsaturated bonds and the type of fillers or modifiers used. A high carbon content may promote particle condensation in the gas phase, resulting in increased smoke production. Furthermore, fillers such as EG, CNT, G and MPP can significantly affect the degradation mechanism, the extent of char formation and the quantity of smoke produced [54,55,56,57].

The results presented in Table 9 indicate that only the PUR composites filled with graphene exhibit a tendency toward reduced smoke production. The composite containing 1 wt.% graphene, PUR-G1, shows a 28.4% reduction in smoke emission, expressed by the D_sMAX_ parameter, relative to the reference PUR sample. Increasing the graphene content in the polymer matrix to 3 wt.% (PUR-G3) leads to an increase in smoke production, reaching a level comparable to that of the unmodified PUR foam. It should also be noted that in the presence of melamine polyphosphate (MPP), the graphene-filled composite again exhibits reduced smoke production, as reflected by the decrease in the D_sMAX_ parameter. The reduction in D_sMAX_, as well as in Ds(4) and VOF_4_ for the PUR-G3-MPP sample, compared with the reference material, results from the catalytic effect of MPP, which promotes crosslinking and cyclisation reactions in the thermal decomposition residue. Through the synergistic action of graphene and the foamed residue formed during MPP decomposition, an insulating carbonaceous layer is produced, effectively limiting the release of gaseous degradation products, including solid carbonaceous particles, into the gas phase.

For the composites containing graphene, particular attention should be paid to the Ds(4) and VOF_4_ parameters. Ds(4) reflects the amount of smoke emitted after four minutes of testing. The data in Table 9 clearly show that the kinetics of smoke production in the graphene-containing composites, as well as in the graphene–MPP system, is significantly lower than in the reference sample. The Ds(4) values correlate well with the VOF_4_ parameter, which represents the volumetric optical density of smoke after 4 min of exposure.

In contrast, for the PUR composites containing expanded graphite (EG) or carbon nanotubes (CNTs), the values of D_sMAX_, Ds(4) and VOF_4_ are considerably higher than those of the reference PUR foam, clearly indicating an increase in smoke production.

The increased smoke emission observed for PUR materials containing EG or CNTs is undoubtedly related to the higher carbon content of the polymer matrix, but more importantly, it results from the fundamentally different mechanisms of action of EG and CNTs compared with graphene. Expanded graphite undergoes intense expansion during the initial stage of thermal decomposition. The expanding carbonaceous structure forms a barrier that is impermeable to both solid and gaseous degradation products. Carbon nanotubes, on the other hand, tend to immobilize within the liquid degradation products of the polymer while forming an insulating carbonaceous layer. Consequently, smoke emission in composites containing EG or CNTs is significantly higher especially at the early stages of thermal decomposition than in the reference material.

### 3.8. Toxicity

**Figure 18:**
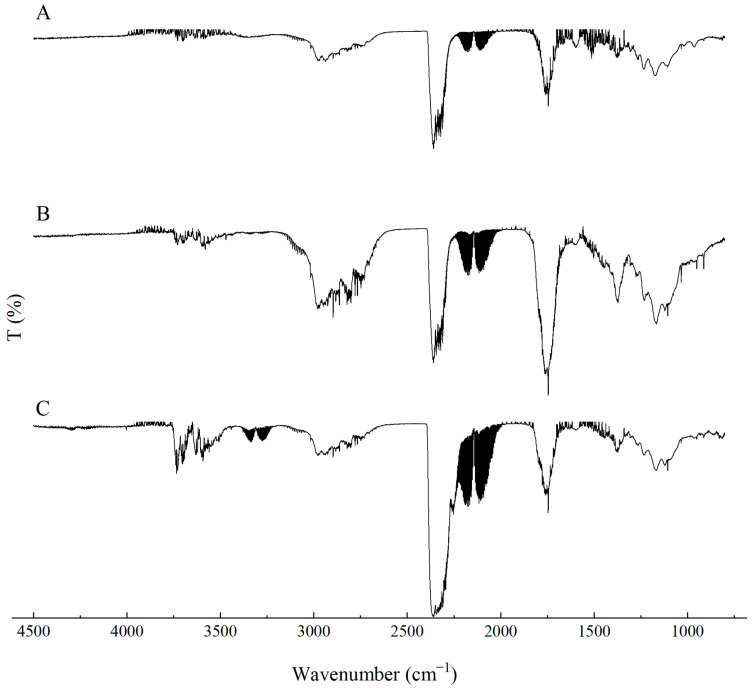
FTIR spectra of gases generated during the thermal decomposition of unmodified PUR using the coupled TG–gas analyser Omega 5 at 350 °C (**A**), 450 °C (**B**) and 550 °C (**C**).

**Figure 19:**
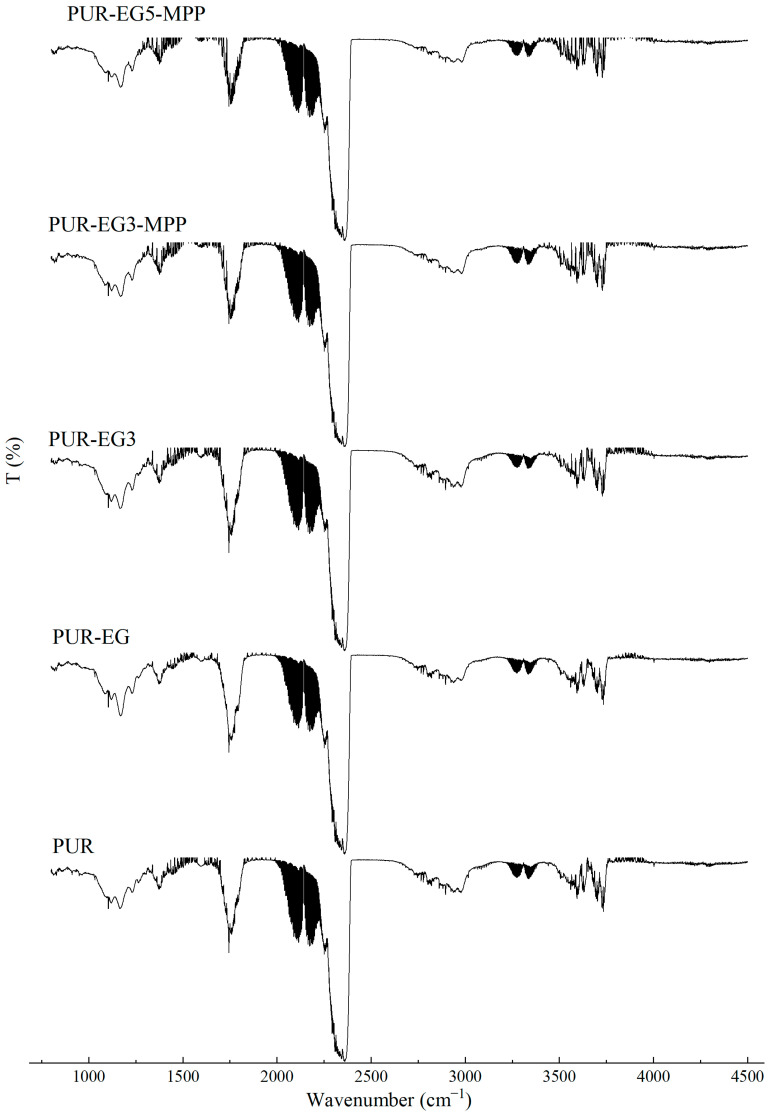
Selected FTIR spectra of gases generated during the thermal decomposition of the composites (550 °C) using the coupled TG–gas analyzer Omega 5.

Figure 18 presents the FTIR spectra of the gaseous products released from the unmodified PUR foam at 350, 450 and 550 °C. The FTIR spectra of the gases evolved during the thermal decomposition of the developed PUR composites show characteristic absorption bands at 1745 cm^−1^ corresponding to carbonyl group vibrations (Figure 19) [58]. All PUR composites exhibit intense absorption bands associated with the emission of CO (2050–2220 cm^−1^), CO_2_ (2285–2380 cm^−1^) and HCN (3228–3388 cm^−1^).

**Figure 20:**
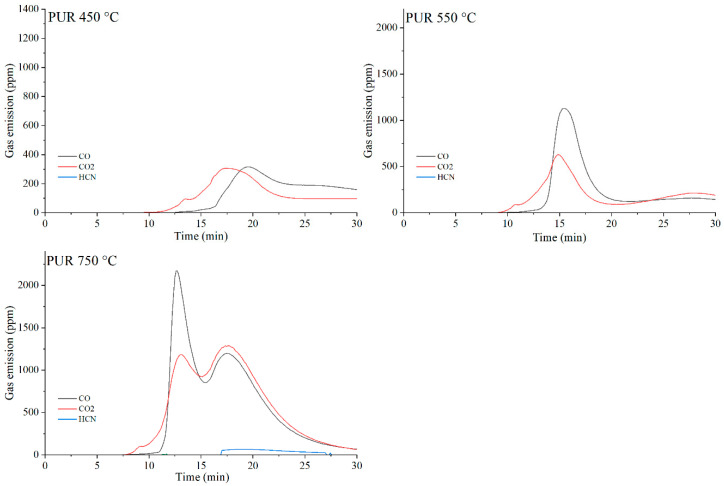
Real-time gas emission profiles of PUR foams at 450, 550 and 750 °C.

**Figure 21:**
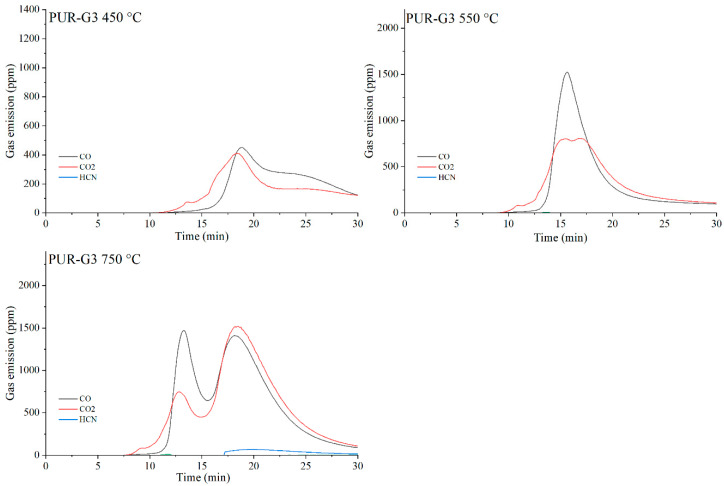
Real-time gas emission profiles of the PUR-G3 composites at 450, 550 and 750 °C.

**Figure 22:**
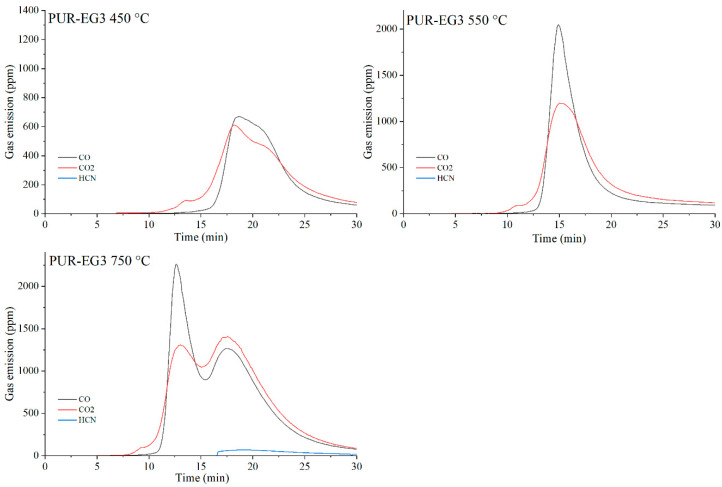
Real-time gas emission profiles of the PUR-EG3 composites at 450, 550 and 750 °C.

**Figure 23:**
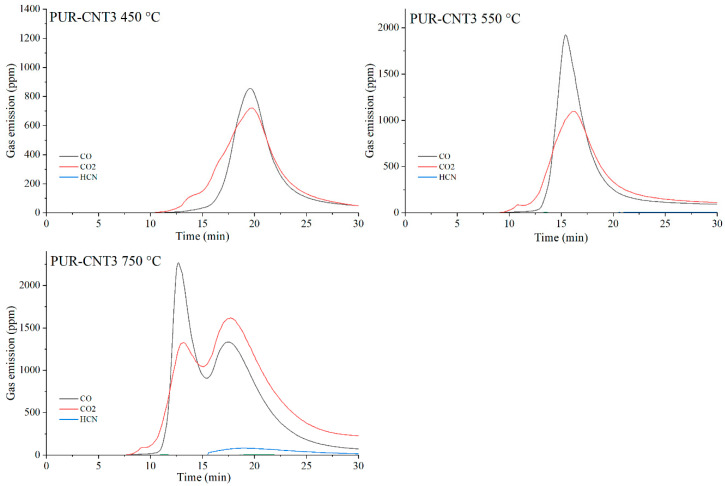
Real-time gas emission profiles of the PUR-CNT3 composites at 450, 550 and 750 °C.

The data presented in Table 10 show that, as expected, the incorporation of carbon-based fillers, whether graphene (G), expanded graphite (EG) or carbon nanotubes (CNTs), into the PUR matrix increases the emission of both CO and CO_2_ during thermal degradation. The rise in CO and CO_2_ concentrations in the gaseous degradation products is proportional to the sampling temperature (Table 10, Figure 20, Figure 21, Figure 22 and Figure 23). A noteworthy observation is the increase in CO_2_ concentration relative to CO for all samples at 750 °C. This shift indicates that, at this temperature, the thermal degradation of the composites is approaching completion (reflected by a decrease in CO release), while simultaneous oxidation of CO to CO_2_ occurs.

The toxicity results correlate well with the smoke emission analysis. It is particularly evident that, especially at 450 °C and at 550 and 750 °C the emissions of CO and CO_2_ are significantly lower for the composites containing graphene than for those filled with EG or CNTs. This behavior arises from both the high thermal stability of graphene and its specific morphology. As a plate-like filler with the highest thermal stability among the carbon additives (Table 4), graphene forms an insulating interfacial barrier during PUR decomposition, which limits heat and mass transfer between the sample and the flame. The effectiveness of this barrier is further demonstrated by the absence of HCN and NO_x_ emissions during the thermal degradation of the PUR-G3-MPP sample at 450 °C. At the same temperature, the PUR-CNT3-MPP composite shows detectable HCN, while the PUR-EG5-MPP sample releases both HCN and NO_x_ (Table 10).

As the temperature of gas sampling increases, both hydrogen cyanide (HCN) and nitrogen oxides (NO_x_) become more prominent in the degradation products. Their presence is primarily associated with the decomposition of the polymer matrix. It is noteworthy that in the presence of MPP particularly at 550 °C the concentration of HCN increases in several composites, such as PUR-G3-MPP and PUR-CNT3-MPP. It is plausible that part of the ammonia released during the thermal decomposition of melamine polyphosphate acts as a precursor to HCN formation.

### 3.9. Analysis of FED Toxicity Indices

Toxicity indices (FED) were evaluated at three temperatures: 450, 550 and 750 °C (Table 11). The Fractional Effective Dose (FED) is defined as the ratio of the amount of each toxic species to the dose that produces a specified physiological effect (incapacitation or lethality). FED30 represents the value of the FED index after 30 min of exposure. If FED30 is below 0.3, the dose is considered safe, and if FED30 is equal to or exceeds 1.0, the combined action of the toxic gases is assumed to incapacitate or kill 50% of the exposed population.

In addition to FED 30, two further threshold parameters are significant: FED 0.3 and FED 1. FED 0.3 indicates the time at which the FED30 curve first exceeds 0.3, while FED_1_ denotes the time at which the FED 30 value exceeds 1.0 (Table 11).

The WLC_50_ index, calculated as the difference between the emission level and the FED_30_ threshold, describes the concentration of gaseous fire effluents (CO and HCN) that would cause 50% lethality within 30 min of exposure. The WLC_50SM_ toxicometric index is defined as the arithmetic sum of WLC_50_ values determined at 350, 450 and 550 °C. Lower WLC_50_ and WLC_50SM_ values correspond to higher toxicity of the gaseous effluents.

The results shown in Table 11 clearly demonstrate that the toxicity of the gaseous degradation products of the reference PUR increases with temperature. At 450 °C the FED30 value is 0.23, which represents a safe dose. Increasing the temperature to 550 °C causes FED30 to exceed the safe threshold of 0.3. Further temperature elevation to 750 °C increases FED30 to 0.78, well above the threshold value. It should also be noted that for the reference PUR, the time required to reach the FED0.3 threshold decreases with increasing temperature. Correspondingly, the WLC_50_ index decreases with temperature, clearly indicating increased toxicity of the gaseous effluents.

Among the tested materials, the highest toxicity expressed by FED30 and WLC_50_ was observed for the composites PUR-EG5-MPP (750 °C), PUR-G3-MPP (750 °C) and PUR-CNT3 (750 °C). These materials also exhibited the shortest time required to reach the FED0.3 threshold.

With increasing decomposition temperature, the relative contribution of CO and HCN also changes. At higher temperatures, the proportion of CO in the CO/HCN mixture increases at the expense of HCN.

The incorporation of carbon fillers also in combined systems with MPP generally increases the toxicity of the PUR composites, as indicated by the reduction in the WLC_50SM_ parameter (Table 11). The increased toxicity of the gaseous degradation products, especially at the highest temperature of 750 °C, results not only from the higher carbon content of the PUR composites but also from the nitrogen donor introduced in the form of MPP.

## 4. Conclusions

This study investigated the influence of selected carbon-based fillers graphene, expanded graphite, carbon nanotubes and their synergistic combinations with melamine polyphosphate (MPP) on thermal stability, flammability, smoke emission and toxicity of fire effluents generated by flexible PUR foams. The comprehensive analysis demonstrated that incorporation of appropriately selected fillers, particularly in optimized proportions, can significantly improve fire-safety-related properties of PUR materials.

The results showed that all applied carbon fillers markedly reduce the dm/dt parameter. This reduction, proportional to the filler content in the polymer matrix, indicates that a smaller amount of volatile, combustible degradation products escapes from the sample into the flame zone. The most pronounced decrease in dm/dt was recorded for PUR-G3 and PUR-EG3-MPP.

Virtually all tested fillers increased the amount of solid residue formed after thermal decomposition (P_TD_). The increase in P_TD_ in the combined carbon-filler/MPP systems follows directly from their mechanism of action: melamine polyphosphate undergoes intense foaming during thermal decomposition, forming a solid protective phase, while carbon fillers act as carbon donors. Additionally, phosphoric acid released from MPP promotes char-forming crosslinking reactions. The combined use of MPP with graphene, expanded graphite or CNTs significantly reduced the flammability of the PUR foams, with the expanded-graphite/MPP system being the most effective among the tested fire-retardant formulations.

The increased smoke emission observed for PUR materials containing EG and CNTs results not only from the higher carbon content of the polymer matrix but also from their markedly different modes of action compared with graphene. Expanded graphite undergoes rapid expansion during the initial stages of thermal decomposition, forming a barrier that is impermeable to both gaseous and solid degradation products. Carbon nanotubes, while forming a carbonaceous insulating layer, become immobilized within the liquid decomposition products of the polymer. As a consequence, CNTs containing composites show significantly higher smoke emission especially in the early stages of decomposition than materials containing EG or the reference PUR.

The results in Table 11 clearly show that the toxicity of gaseous degradation products from the reference PUR sample increases with decomposition temperature. The introduction of carbon fillers, including their synergistic combinations with MPP, generally increases the toxicity of the PUR composites, as evidenced by the reduction in the WLC_50_SM parameter. The increased toxicity of fire effluents, particularly at elevated temperatures (T = 750 °C), is attributed both to the higher carbon content of the PUR composites and to the presence of nitrogen donors introduced via MPP.

## Figures and Tables

**Table 1:** Component analysis of PUR composites.

Composite	G	EG	CNTs	MPP
PUR-G1	1			
PUR-G3	3			
PUR-G3-MPP	3			1
PUR-EG1		1		
PUR-EG3		3		
PUR-EG3-MPP		3		1
PUR-EG5-MPP		5		1.5
PUR-CNT1			1	
PUR-CNT3			3	
PUR-CNT3-MPP			3	1

**Table 2:** EDS elemental composition of the carbon-based fillers.

Element	Atomic (%)	Atomic Error (%)	Weight (%)	Weight Error (%)	Net Counts
EG					
C	76.0	0.3	67.8	0.3	943,604
O	20.8	0.1	25.2	0.2	111,650
H	3.1	0.0	6.9	0.1	48,425
CNTs					
C	97.8	0.4	89.4	0.4	823,744
H	2.2	0.0	8.5	0.2	25,888
G					
C	99.8	0.4	99.6	0.4	679,642
H	0.2	0.0	0.4	0.0	1085

**Table 3:** Elemental composition of the PUR composites.

Element	Atomic (%)	Atomic Error (%)	Weight (%)	Weight Error (%)	Net Counts
PUR					
C	53.4	0.3	46.7	0.3	69,810
N	7.5	0.7	7.7	0.8	2386
O	39.1	0.5	45.6	0.6	20,102
PUR-EG1					
C	51.1	0.3	44.4	0.2	127,464
N	8.0	0.5	8.1	0.5	4895
O	41.0	0.4	47.5	0.5	40,516
PUR-EG3					
C	55.0	0.3	48.4	0.2	125,807
N	7.7	0.6	7.9	0.6	4117
O	37.3	0.4	43.7	0.5	32,757
PUR-EG3-MPP					
C	54.3	0.2	47.7	0.2	423,542
N	7.6	0.3	7.8	0.3	13,886
O	38.1	0.3	44.5	0.3	114,693
PUR-G1					
C	57.4	0.3	50.9	0.2	148,230
N	8.1	0.6	8.4	0.6	4700
O	34.5	0.4	40.7	0.4	33,266
PUR-G3					
C	53.6	0.3	47.0	0.2	121,221
N	8.6	0.6	8.8	0.6	4602
O	37.8	0.4	44.2	0.5	32,864
PUR-G3-MPP					
C	53.2	0.2	45.3	0.2	456,134
N	6.3	0.3	6.3	0.3	14,065
O	37.3	0.2	42.4	0.3	139,565
PUR-CNT1					
C	54.0	0.3	46.6	0.2	142,829
N	7.3	0.5	7.4	0.5	4889
O	36.9	0.4	42.4	0.4	40,544
H	1.8	0.1	3.7	0.2	2746
PUR-CNT3					
C	52.1	0.2	43.4	0.2	199,717
N	6.0	0.4	5.8	0.4	6520
O	36.8	0.3	40.8	0.3	66,599
H	5.1	0.1	10.0	0.2	12,491
PUR-CNT3-MPP					
C	52.9	0.2	46.9	0.2	242,803
N	7.1	0.4	8.2	0.4	8939
O	36.0	0.3	42.8	0.4	66,930
H	4.1	0.1	8.3	0.2	8147

**Table 4:** Thermal parameters obtained for the fillers used in the PUR composites.

Flame Retardant	T_5_ (°C)	T_50_ (°C)	T_RMAX_ (°C)	dm/dt (%/min)	P_TD_ (%)	P_600_ (%)	P_900_ (%)
G	640	740	753	7.4	9.2	99.8	9.1
EG	185	750	193	13.6	70.8	69.6	10.2
CNTs	515	555	550	32.4	3.2	3.5	2.3
MPP	383	570	390	4.2	27.6	46.7	22.4

**Table 5:** Thermal parameters of the PUR composites.

Composite	T_5_ (°C)	T_50_ (°C)	T_RMAX_ (°C)	dm/dt (%/min)	P_TD_ (%)	∆T_s_ (°C)	P_600_ (%)
PUR	301	364	368	15	27.3	430–600	2.8
PUR-G1	293	359	353	10.9	24.6	420–600	2.5
PUR-G3	291	376	343	8	30.1	430–600	4.7
PUR-G3-MPP	304	377	376	10.5	32.8	435–600	6.2
PUR-EG1	296	369	356	9.6	30.1	425–600	4.9
PUR-EG3	297	366	346	9.7	28.2	435–600	4.8
PUR-EG3-MPP	297	381	378	8.1	34.6	430–600	6.6
PUR-EG5-MPP	278	386	329	10.6	48.9	396–600	8.2
PUR-CNT1	304	371	351	8.9	30.1	425–600	3.5
PUR-CNT3	299	367	371	12.5	27.9	430–600	1.0
PUR-CNT3-MPP	303	369	366	12.3	32.6	440–600	7.3

**Table 6:** Flammability results of the PUR composites PUR, PUR-G1, PUR-G3, PUR-EG1, PUR-EG3, PUR-CNT1 and PUR-CNT3 obtained using cone calorimetry.

Composite	PUR	PUR-G1	PUR-G3	PUR-EG1	PUR-EG3	PUR-CNT1	PUR-CNT3
t_i_ (s)	54	25	19	20	12	17	12
_tf-0_ (s)	455	499	674	715	1491	494	700
HRR_max_ (kW/m^2^)	318.04	271.37	229.38	245.00	112.80	315.55	246.52
HRR (kW/m^2^)	218.86	192.20	159.13	147.93	73.55	201.69	174.71
EHC_max_ (MJ/kg)	79.35	73.89	77.90	76.03	78.48	78.55	77.36
EHC (MJ/kg)	29.71	31.07	35.37	34.75	38.87	30.90	35.87
MLR_max_ (g/s)	0.16	0.13	0.11	0.11	0.06	0.15	0.11
MLR (g/s)	0.06	0.05	0.04	0.04	0.02	0.06	0.04
AMLR (g/m^2^·s)	9.86	7.95	5.35	6.07	2.01	8.70	5.91
THR (MJ/m^2^)	87.70	90.70	103.90	103.00	108.80	95.30	119.80
FIGRA (kW/m^2^·s)	1.20	0.89	0.59	1.14	1.33	1.11	0.90
MARHE (kW/m^2^)	198.36	185.85	164.66	168.75	81.17	202.18	189.50

**Table 7:** Flammability results of the PUR composites PUR, PUR-G3-MPP, PUR-G5-MPP, PUR-EG3-MPP, PUR-EG5-MPP and PUR-CNT3-MPP obtained using cone calorimetry.

Composite	PUR	PUR-G3-MPP	PUR-EG3-MPP	PUR-EG5-MPP	PUR-CNT3-MPP
t_i_ (s)	54	19	13	10	18
t_f-0_ (s)	455	553	1165	154	597
HRR_max_ (kW/m^2^)	318.04	215.28	92.32	81.19	219.88
HRR (kW/m^2^)	218.86	157.18	73.74	52.21	153.15
EHC_max_ (MJ/kg)	79.35	77.99	79.20	74.80	78.56
EHC (MJ/kg)	29.71	29.42	31.20	18.01	30.46
MLR_max_ (g/s)	0.16	0.14	0.12	0.06	0.12
MLR (g/s)	0.06	0.05	0.02	0.03	0.04
AMLR (g/m^2^·s)	9.86	6.53	2.59	3.45	5.99
THR (MJ/m^2^)	87.70	83.80	84.90	7.4	88.40
FIGRA (kW/m^2^·s)	54	19	13	10	0.85
MARHE (kW/m^2^)	455	553	1165	154	154.52

**Table 8:** Flammability results of the PUR composites obtained using PCFC microcalorimetry.

Composite	HRR_MAX_ (W/g)	THRR_MAX_ (°C)	THR (kJ/g)	HRC (J/gK)
PUR	552.6	408	22.8	529
PUR-G1	608	416	24.7	593
PUR-G3	541	409	23.3	523
PUR-G3-MPP	388	416	21.4	376
PUR-EG1	584	410	24.3	565
PUR-EG3	514	410	23.1	497
PUR-EG3-MPP	375	410	21.6	361
PUR-EG5-MPP	341	405	21.6	322
PUR-CNT1	560	409	23.7	545
PUR-CNT3	558	418	22.4	539
PUR-CNT3-MPP	389	414	20.5	373

**Table 9:** Optical smoke density analysis of the PUR composites.

Composite	D_sMAX_	TD_sMAX_	Ds(4)	VOF_4_
PUR	203.8	598 s	74.22	173.8
PUR-G1	145.9	600 s	54.63	85.6
PUR-G3	205.6	600 s	69.75	110.1
PUR-G3-MPP	166.3	600 s	56.95	94.35
PUR-EG1	230	600 s	83.88	112.6
PUR-EG3	228.8	590 s	121.1	191.8
PUR-EG3-MPP	260.8	415 s	225.8	385.9
PUR-EG5-MPP	243.5	305 s	236.1	477.4
PUR-CNT1	217.6	600 s	77.72	104.6
PUR-CNT3	305.1	592 s	113.4	137.6
PUR-CNT3-MPP	329.2	598 s	153.9	195.9

**Table 10:** Maximum concentrations of gases released from PUR composites expressed in mg/m^3^.

Composite	CO_2_	CO	HCN	NO_x_
450 °C				
PUR	601	395	-	-
PUR-G3	807	564	-	-
PUR-G3-MPP	770	426	-	-
PUR-EG3	1202	837	-	-
PUR-EG3-MPP	1363	970	-	-
PUR-EG5-MPP	2162	1663	13	8
PUR-CNT3	1416	1068	-	-
PUR-CNT3-MPP	1488	1232	10	-
550 °C				
PUR	1233	1408	-	-
PUR-G3	1588	1907	-	16
PUR-G3-MPP	1255	1515	12	14
PUR-EG3	2354	2561	-	-
PUR-EG3-MPP	1940	1752	-	-
PUR-EG5-MPP	2739	2724	23	12
PUR-CNT3	2154	2406	10	12
PUR-CNT3-MPP	2134	2026	17	12
750 °C				
PUR	2531	2716	81	21
PUR-G3	2981	1837	82	21
PUR-G3-MPP	4353	2489	90	18
PUR-EG3	2767	2829	83	-
PUR-EG3-MPP	3542	2156	88	21
PUR-EG5-MPP	3273	2932	92	16
PUR-CNT3	3183	2834	99	18
PUR-CNT3-MPP	4002	2251	98	21

**Table 11:** Toxicity of gaseous thermal decomposition products of PUR composites.

Composite	FED 0.3 (min)	FED 1.0 (min)	FED30	WLC_50_ (g/m^3^)	CO/HCN% (FED30)	WLC_50SM_
PUR-450 °C	-	-	0.23	15.70	39/61	12.49
PUR-550 °C	30	-	0.31	14.76	54/46	
PUR-750 °C	17	-	**0.78**	7.02	74/26	
PUR-EG3 450 °C	-	-	0.28	13.94	50/50	10.29
PUR-EG3 550 °C	19	-	0.42	10.75	66/34	
PUR-EG3 750 °C	16	-	0.86	6.18	78/22	
PUR-EG3-MPP 450 °C	-	-	0.27	13.68	48/52	16.34
PUR-EG3-MPP 550 °C	26	-	0.34	12.25	58/42	
PUR-EG3-MPP 750 °C	17	-	**0.78**	6.76	76/24	
PUR-EG5-MPP 450 °C	29	-	0.31	12.58	54/46	9.23
PUR-EG5-MPP 550 °C	18	-	0.45	9.69	68/32	
PUR-EG5-MPP 750 °C	15	-	**0.98**	5.42	80/20	
PUR-G3 450 °C	-	-	0.26	14.82	44/56	11.14
PUR-G3 550 °C	22	-	0.38	11.93	62/38	
PUR-G3 750 °C	18	-	**0.79**	6.69	76/24	
PUR-G3-MPP 450 °C	-	-	0.25	14.69	43/57	10.86
PUR-G3-MPP 550 °C	25	-	0.37	11.40	61/39	
PUR-G3-MPP 750 °C	19	-	**0.82**	6.50	76/24	
PUR-CNT3 450 °C	-	-	0.28	14.03	49/51	10.24
PUR-CNT3 550 °C	20	-	0.41	11.03	65/35	
PUR-CNT3 750 °C	16	-	**0.97**	5.66	78/22	
PUR-CNT3-MPP 450 °C	-	-	0.30	12.85	51/49	9.44
PUR-CNT3-MPP 550 °C	21	-	0.41	10.31	64/36	
PUR-CNT3-MPP 750 °C	16	29	**1.03**	5.17	79/21	

## Data Availability

The original contributions presented in this study are included in the article. Further inquiries can be directed to the corresponding authors.
